# A comparison of medically serious suicide attempters admitted to intensive care units versus other medically serious suicide attempters

**DOI:** 10.1186/s12888-022-04427-8

**Published:** 2022-12-19

**Authors:** Marta Quesada-Franco, Mª Dolores Braquehais, Sergi Valero, Anna Beneria, J. A. Ramos-Quiroga, Enrique Baca-García, Luis Pintor-Pérez

**Affiliations:** 1 Department of Psychiatry, Hospital Universitari, Vall d’Hebron, Barcelona, Spain; 2 Department of Psychiatry and Legal Medicine, Universitat Autònoma de Barcelona, Barcelona, Spain; 3 Psychiatry, Mental Health and Addictions Research Group, Vall d’Hebron Research Institute (VHIR), Barcelona, Spain; 4 Integral Care Programme for Sick Health Professionals, Galatea Clinic, Barcelona, Spain; 5 Biomedical Network Research Centre On Mental Health (CIBERSAM), Barcelona, Spain; 6 School of Medicine, Universitat Internacional de Catalunya (UIC), Barcelona, Spain; 7 Ace Alzheimer Center Barcelona, Universitat Internacional de Catalunya, Barcelona, Spain; 8 Networking Research Center on Neurodegenerative Diseases (CIBERNED), Instituto de Salud Carlos III, Madrid, Spain; 9 Department of Psychiatry, University Hospital Jimenez Diaz Foundation, Madrid, Spain; 10 Department of Psychiatry, University Hospital Rey Juan Carlos, Mostoles, Spain; 11 Department of Psychiatry, General Hospital of Villalba, Madrid, Spain; 12 Department of Psychiatry, University Hospital Infanta Elena, Valdemoro, Spain; 13 Department of Psychiatry, Madrid Autonomous University, Madrid, Spain; 14 CIBERSAM (Centro de Investigacion en Salud Mental), Carlos III Institute of Health, Madrid, Spain; 15 UniversidadCatolica del Maule, Talca, Chile; 16 Department of Psychiatry, Centre Hospitalier Universitaire de Nîmes, Nimes, France; 17 Department of Psychiatry, Hospital Clinic, CIBERSAM, Universitat de Barcelona, Barcelona, Spain

**Keywords:** Medically serious suicide attempts, Nearly lethal suicide attempts, Intensive care unit

## Abstract

**Background:**

Medically serious suicide attempts (MSSA) represent a subgroup of clinically heterogeneous suicidal behaviours very close to deaths by suicide. A simple definition of an MSSA is a suicide attempt with life-threatening consequences, regardless of the severity of the attempter’s mental disorder. Few studies have specifically analysed the heterogeneity of MSSA. Therefore, the aim of this study is to describe the profile of individuals who made a highly severe MSSA and to compare those admitted to Intensive Care Units (ICU) – including Burn Units– with other MSSA admitted to other medical and surgical units.

**Methods:**

The study sample consisted of 168 patients consecutively admitted to non-psychiatric wards from two public hospitals in Barcelona after an MSSA during a 3-year period. In order to select more severe MSSA, the minimum hospital stay was expanded from Beautrais’ definition of ≥ 24 h to ≥ 48 h. Mean hospital stay was 23.68 (SD = 41.14) days. Patients needing ICU treatment (*n* = 99) were compared to other MSSArs (*n* = 69) that were admitted to other medical and surgical units, not requiring intensive care treatment, with an initial bivariant analysis followed by a logistic regression analysis using conditional entrance.

**Results:**

Medically serious suicide attempters (MSSArs) spent more time hospitalized, more frequently reported recent stressful life events, were more likely to have at least one prior suicide attempt (SA) and their current attempt was more frequently non-planned, compared to the profile of MSSArs reported in previous studies. The most frequent method was medication overdose (67.3%) and jumping from heights (23.2%). Among those who chose more than one method (37.6%), the most frequent combination was medication overdose and drug use. Affective disorders and personality disorders were the most frequent diagnoses. Higher educational level, history of previous mental disorders and prior lifetime suicide attempts were significantly more frequent among those admitted to ICU compared to other MSSArs. Patients needing admission to ICU less frequently used self-poisoning and cuts.

**Conclusions:**

MSSA needing ICU admission can be regarded clinically as similar to attempts resulting in suicide. More research on this type of highly severe suicide behaviour is needed due to its serious implications both from a clinical and public health perspective.

## Background

More than 700,000 people die by suicide every year, which is one person every 40 s and accounted for 1.3% of all deaths worldwide in 2019 [1]. Suicide occurs at all stages of life, being the fourth cause of death in people between 15 and 29 years [1].

Individuals who made an MSSA in the past are at higher risk of dying by suicide than those who make less serious lethal suicide attempts [2, 3]. Studies have shown that survivors of MSSA are epidemiologically very similar to those who die by suicide [4, 5]. Consequently, recent research has focused on subjects with MSSA, as the study of this subgroup can best shed light on deaths by suicide [3]. Moreover, from the methodological perspective, the study of MSSA overcomes some problems related to the study of suicidal behaviour. In contrast to the psychological autopsy conducted in suicides, in MSSA the main source of information is the survivor.

However, criteria to define an MSSA are heterogeneous. Broadly, a simple definition is suicide attempts with life-threatening consequences, regardless of the severity of the attempter’s mental disorder. Beautrais and collaborators provide a specific clinical definition of MSSA: patients who require hospital admission for more than 24 h after the attempt and met one of the following treatment criteria: a) treatment in a specialized clinical unit (i.e.: intensive care, hyperbaric or burn units), c) need of surgery under general anaesthesia, c) need of medical treatment beyond gastric lavage, activated charcoal, and/or routine neurological observations; and, d) patients who require hospital admission for more than 24 h not because of the aforementioned criteria but because of the highly lethal suicidal methods (e.g.: hanging or gunshot) [4, 6–11].

Other studies define MSSA with psychometric scales such as: the *Self-inflicted Injury Severity* (SIISF) [12–14]; *the Lethality Scale* [15]; the *Lethality of Suicide Attempt Scales II (LSARS-II)* [16, 17]; the *Lethality Rating Scale (LRS)* [3, 18–21] or the *Risk Rescue Rating Scales (RRRS)* [22–24]. Other researchers refer to the time spent in non-psychiatric wards after the MSSA. For instance, in certain studies the mean hospital length of stay was 19 days [3, 20, 21] and 22.7 days in other studies [25].

Aside from the medical consequences of the MSSA, from a psychosocial analysis they can be conceptualized as a heterogenous group [26]. A cluster analysis study based on social-demographic and clinical factors conducted with 124 MSSA found that three major groups of patients could be identified: 1) “impulsive-ambivalent”, predominantly females, with a less severe suicide attempt, generally after a personal crisis and with low scores on anxiety and depression; 2) “marked intent”, predominantly males, with more lethal attempts compared to the first group, more violent suicidal methods (self-poisoning by pesticide and gas), and greater prevalence of moderate and severe depressive disorders; and, 3) “aborted suicides”, with a higher risk of becoming deaths by suicide: they could have died if there had not been a medical intervention because their lethal means was even more violent compared to the second group (firearms, hanging, being run over, etc.) and with the greatest prevalence of severe depressive disorder. Therefore, distinguishing between several clinical presentations of patients admitted to a general hospital after an MSSA is a step beyond the simplistic notion that this subset of suicide attempters is a homogeneous clinical group [26].

The characterization of MSSA subgroups according to the severity of the medical consequences of the attempt may help elucidate the heterogeneity of these nearly lethal suicide attempts. This is the main reason why we decided to compare the profile of patients with MSSA who needed specialized medical care to other MSSA. We chose a more restrictive definition of MSSA, expanding Beautrais’ minimum hospital stay of ≥ 24 [4, 6–11] to ≥ 48 h, in order to identify the most severe MSSA. The concept of MSSA focuses on the medical consequences of the attempt, regardless of its psychiatric severity.

Our main hypothesis was that there would be significant socio-demographic and clinical differences between MSSA needing ICU admission and those who did not. This is also the first study conducted in Spain on MSSA in urban areas and the characteristics of this suicidal subgroup are compared to findings from other studies on MSSA conducted to date.

Patients requiring admission to intensive care units (ICU) for the consequences of suicide attempts can be considered as those who come closest to dying [27, 28]. Thus, being able to identify the profile of patients whose attempts are nearly fatal could help develop more effective suicide prevention strategies. Moreover, in addition to the suffering and possible longer-term consequences faced by the individual patients, ICU admissions have a higher financial cost in comparison to those to other units [29].

## Method

### Study design

This cross-sectional study was conducted over a three-year period at two tertiary university affiliated general hospitals, with all the medical and surgical specialities and services required for patients with unusually severe, complex, or uncommon health problems [30]. Both public hospitals are located in the metropolitan area of Barcelona (Spain). Valld’Hebron University Hospital and Clinic Hospital provide medical coverage to catchment areas covering a population of 400,000 and 540,000 people respectively. In Spain, due to the universal coverage of its public health system, the majority of patients with MSSA are initially referred to public hospitals in order to stabilize their medical situation.

**Fig. 1:**
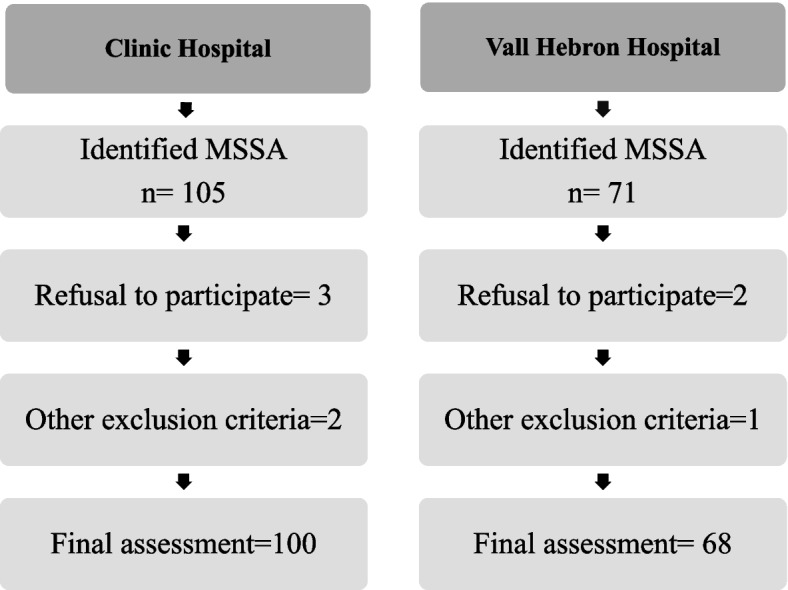
Study flow-chart. MSSA: Medically serious suicide attempts (needing to be hospitalized at a medical/surgical unit ≥ 48 h after the attempt)

The study group consisted of one hundred and sixty-eight patients consecutively admitted to the emergency units of both hospitals after an MSSA. Patients from Clinic Hospital (*n* = 100) were included from February 2007 to August 2010 while those from Vall d’Hebron University Hospital (*n* = 68) were included from February 2010 to October 2012 (see Fig. 1).

### Subjects

The sample consisted of 168 adults with MSSA admitted to the Emergency Care Units of both hospitals. The definition of MSSA followed all Beautrais’ inclusion criteria [4, 6–11], but hospital admission was extended from Beautrais’ 24 h to ≥ 48 h, in order to have a more restrictive inclusion criterion with respect to the severity of the attempt (see Table 1). We selected MSSA admitted to specialized care units (including Intensive Care and Burn Units) because of the serious medical consequences of their suicide attempt and compared them to other medically serious suicide attempters admitted in other medical and surgical wards and not requiring intensive care treatment in ICU.

**Table 1:** Inclusion and exclusion criteria

Inclusion Criteria	Exclusion Criteria
• ≥ 18 years old • Admitted via the Emergency Unit after a medically serious suicide attempt (MSSA) • Need of hospital admission ≥ 48 h • Definition of MSSA: at least 1 of the following: ✓ Need of specialized medical care unit ✓ Need of surgery (superficial cuts excluded) ✓ Medical treatment beyond basic gastric lavage, activated charcoal and/or basic neurological assessment ✓ Highly lethal suicide methods with a high risk of fatality, especially hanging or gunshot who were hospitalized for ≥ 48 h, but did not meet the preceding criteria • Informed consent	• Current moderate/severe cognitive impairment • Current severe psychomotor agitation • Patients admitted to the hospital for other reasons (not for a suicide attempt) • Refusal to sign informed consent

### Procedure and assessment

MSSA were identified daily on Emergency Room databases. In addition to medical assistance, psychiatric assessment of MSSA is always performed during hospitalization once the medical condition is sufficiently stabilized. Researchers involved in the study evaluated the patient during the first week after the MSSA. It was independent of clinical assistance and provided by expert psychiatrists during hospitalization.

Psychosocial, clinical and suicidiological variables related to suicide risk and protective factors [31] were recorded using an ad hoc questionnaire that included:

- ∙ Social-demographics: age, sex, nationality, marital status, educational level, employment situation, and legal problems.
- ∙ Physical and/or sexual abuse during their childhood or/and adulthood.
- ∙ Stressful life events the year prior to the Index Suicide Attempt (ISA).
- ∙ Current medical morbidity (not related to the ISA).
- ∙ Psychiatric history: method of current SA; presence of SA in the 5 years prior to the ISA; previous psychiatry emergency room or inpatient admissions; previous outpatient psychiatric or psychological treatment; and, family history of psychiatric disorders and suicide attempts.

Lethality of the attempt was evaluated using a simple clinical criterion: those needing ICU treatment after the suicide attempt versus those MSSA whose medical consequences were not so severe. Diagnosis was conducted according to DSM-IV-TR criteria [32]. Psychometric assessments included:

- ∙ The validated Spanish-language version of Beck’s Hopelessness Scale (BHS) [33, 34] is composed of 20 items that can be defined as true or false, evaluating the scope of negative expectations about the immediate and long-term future. The items that indicate hopelessness score 1 point, while those that do not indicate it score 0 points. The number of points measure the severity of hopelessness: 0–3 is minimum or normal, 4–8 is mild, 9–14 is moderate and 15–20 is severe.
- ∙ The validated Spanish-language version of Barratt’s Impulsiveness Scale (BIS-11) [35] is composed of 30 self-report items scored between 0 to 4 (range of total score 0–120), with cognitive, motor and non-planning impulsiveness subscales.
- ∙ The validated Spanish-language version of Beck’s Suicidal Intent Scale (SIS) [36, 37] is composed of 15 items, with the first eight items measuring objective aspects of suicide intent (including isolation, time, acting to get help, and suicide note) and the last seven items measuring patients’ self-reports of intent (such as alleged purpose, expectations of fatality, and attitude toward dying) It is rated by summing up the scores on each item (varying from 0 to 2), and the final score ranges from 0 to 30 points. In the scale's validation studies, the mean scores were 16.3 for highly serious suicide attempts; 10.1 for medium severity, and 6.7 for low severity.

The SIS measures the individual’s intent to die by any suicide attempt. The scales were selected according to pre-existing evidence on a brief screening of suicidal behaviours [2, 18, 20, 38] in order to capture the most important information regarding the suicide attempt, whilst also considering that the in situ evaluation was limited by the patient's condition.

### Statistics

Bivariate and multivariate analyses were executed. First, a descriptive analysis of all variables as percentages, means, and standard deviations was conducted. To compare patients admitted in ICU with other MSSA patients, odds ratio with 95% confidence intervals were used to analyse the relationship between binary variables. Bonferroni correction for multiples testing was used in variables found statistically significant at bi-variate analysis, in order to minimize the type I error. Finally, logistic regression analyses were conducted using the variables that remained statistically significant after the Bonferroni correction. In order to avoid co-linearity related variables were not included in the multivariate analyses. The dependent variable was need of ICU (0 = non-ICU and 1 = ICU). A conditional entrance method was used to select variables in the model. All statistical hypotheses were two-sided and conducted with an alpha of 0.05. All analyses were performed using the SPSS version 20.

## Results

The sample included 168 patients with a mean age of 45 (SD = 17.21) years. Males were less prevalent than women (47.6%, *n* = 80), with 38% (*n* = 64) only achieving elementary education and almost a quarter of the sample (20%, *n* = 34) living alone. Physical abuse was reported by 13.7% (*n* = 23), while 7.1% (*n* = 12) had a history of sexual abuse during their childhood/adolescence. Stressful life events were reported by 92% (*n* = 154) the year prior the MSSA, with most of them reporting serious ongoing interpersonal difficulties (73.8%, *n* = 124) while 32.1% (*n* = 54) reported work-related problems. Physical illnesses before the attempt were present in 56.5% (*n* = 95) of the sample, the majority of which were cardiovascular diseases.

MSSA were initially admitted to the Emergency Room (69.6% to the Medical Unit and 30.4% to the Surgical Unit) and later referred to: 1) Intensive Care Units (56.0%); 2) Internal Medicine Hospitalization (28%); 3) Orthopaedics / Trauma Units (10.7%); and, 4) Burn Units (3%). Mean length of hospital stay was 23.68 (DT = 41.14) days. After hospitalization, most patients were discharged to home (61.9%), but 29.8% required psychiatric admission and 6.0% needed to be referred to a rehabilitation hospital.

With respect to their psychiatric history, 90.5% (*n* = 152) of the subjects had a previous psychiatric disorder, 62.5% (*n* = 105) had at least one prior suicide attempt and 42.3% (*n* = 71) of the sample required temporary hospital admission after a previous suicide attempt. The majority (98.8%) of the sample currently met diagnostic criteria for at least one mental disorder: affective disorders (41.9%), personality disorders (41.9%) and substance use disorders (36.9%). Concurrent mental disorders were present in 43% (*n* = 72) of the sample and 41% (*n* = 65) met criteria for an Axis I and Axis II diagnosis.

**Fig. 2:**
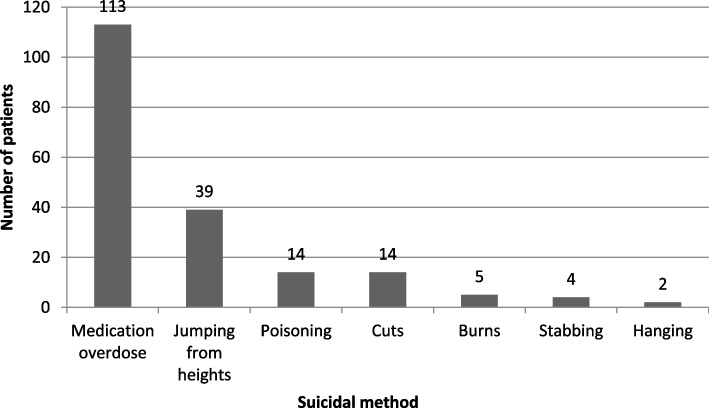
Type of suicide method

With regards to the features of their MSSA, 36.3% used violent suicide means (i.e., jumping from heights, hanging, stabbing, burns and cuts). The different types of suicide methods are depicted in Fig. 2.

The majority of them only used one method (62.5%, *n* = 105), 31% (*n* = 52) combined two, and the rest used three or more methods (6.6%, *n* = 11). Alcohol was present in 26.2% (*n* = 44) and 37.6% (*n* = 61) reported drug use before the attempt. While 43.5% (*n* = 73) reported that they had previously planned the attempt, 40.5% (*n* = 68) answered they had considered suicide less than 3 h before the attempt. Differences in social-demographic and clinical variables between groups are depicted in Table 2.

**Table 2:** Differences in socio-demographic and clinical variables

Socio-demographic variables					
	**ICU MSSArs (*n* = 99)**	**Other MSSArs (*n* = 69)**	**Statistics**		
**Qualitative variables**	**n (%)**	**n (%)**	**OR**		**95% CI**
**Sex** *(male)*	48 (48.5%)	32 (46.4%)	1.09		0.59–2.01
**Immigrant**	13 (13.3%)	9 (13.2%)	1.00		0.4–2.50
**Living alone**	19 (19.2%)	15 (21.7%)	0.86		0.4–1.83
**High educational qualifications**	65 (66.3%)	35 (50.7%)	**1.91**		**1.02–3.60**
**Active working status:**					
- *Unemployed*	15 (16%)	6 (8.8%)	1.88		0.62–5.65
* - Disability/work leave/retired*	51 (54.3%)	41 (60.3%)	0.93		0.46–1.88
* - Active*	28 (29.8%)	21 (30.9%)	Reference		Reference
**Physical and/or sexual abuse**					
* (Childhood / adolescence)*	19(21.1%)	10 (15.4%)	1.47		0.63–3.42
** Stressful life events the year prior to the ISA**	89 (90%)	65 (94%)	0.55		0.16–1.82
** Quantitative variables**	**Mean (SD)**	**Mean (SD)**			
** Age** *(years)*	44.42(16.9)	47.80 (17.59)	0.99	0.97–1.01	
**Clinical variables**					
** Personal mental health history**	95 (96%)	55 (79.7%)	**6.05**	**1.89–19.28**	
** Family mental health history**	59 (62.8%)	39 (59.1%)	1.17	0.61–2.22	
** Chronic medical condition**	48 (48.5%)	41 (59.4%)	0.64	0.65–1.20	
** Acute medical condition**	8 (8.1%)	9 (13%)	0.59	0.21–1.60	
**Discharge**					
* Further hospitalization*	36 (36.4%)	28 (40.6%)	0.58	0.45–1.57	
** Inpatient treatment in prior 12 months**^**a**^	30 (30.3%)	16 (23.2%)	1.44	0.71–2.91	
** Outpatient treatment in prior 12 months**^**a**^	72 (72.7%)	43 (62.3%)	1.61	0.84–3.11	
**Current mental disorders*-IV-TR Diagnoses***					
* Substance use disorder*	35 (35.4%)	27 (39.1%)	0.85	0.45–1.61	
* Mild cognitive disorder*	10 (10.1%)	2 (2.9%)	3.76	0.80–17.75	
* Psychotic disorder*	13 (13.1%)	13 (18.8%)	0.65	0.28–1.51	
* Affective disorder*	38 (38.4%)	31 (44.9%)	0.76	0.41–1.42	
* Personality disorder*	44 (44.4%)	26 (37.7%)	1.27	0.68–2.38	
* Adjustment disorder*	29 (29.3%)	20 (29%)	1.04	0.52–2.06	
** Quantitative variables**	**Mean (SD)**	**Mean (SD)**			
** Length of hospital stay** *(days)*	27.57 (32.39)	18.10 (50.9)	1.01	0.99–1.02	

^a^Psychiatric treatment; *OR* Odds ration, *CI* Confidence Interval, *MSSArs* Medically serious suicide attempters, *ISA* Index Suicide Attempt, *ICU* Intensive Care Units

Higher educational level, history of previous psychiatric disorders and of previous suicide attempts were more frequent among those admitted to ICU. After Bonferroni’s correction, individual history of psychiatric disorder and type of current suicidal method of the MSSA remained statistically significant.

No statistically significant differences between ICU and non-ICU attempters were found when comparing the mean scores of the BIS-11 (motor, non-planning and cognitive subscales as well as the global score), the SIS (conception, preparation, precautions and communication subscales, and global scores) and the Beck’s Hopelessness scale (Table 3).

**Table 3:** Differences in past and current suicide behaviour

	**ICU MSSArs (*n* = 99)** *n (%)*	**Other MSSArs (*n* = 69)** *n (%)*	**Statistics**	
			*OR*	*IC 95%*
**Family history of suicide behaviour**	26 (25.5%)	20 (32.8%)	0.76	0.98–1.51
**Previous suicide attempts**	69 (69.7%)	37 (53.6%)	**1.99**	**1.05–3.77**
**Current serious suicide attempt**				
*** Violent means***	39 (39.4%)	22 (31.9%)	1.39	0.73–2.65
*** Medication overdose***	70 (70.7%)	43 (62.3%)	1.46	0.76–2.80
*** Poisoning***	2 (2%)	12 (17.4%)	**0.10**	**0.02–0.45**
*** Jumping***	28 (28.3%)	11 (15.9%)	2.08	0.95–4.53
*** Hanging***	2 (2%)	0 (0%)	^*^	^*^
*** Cuts***	3 (3%)	11 (15.9%)	**0.17**	**0.04–0.62**
*** Stabbing***	2 (2%)	2 (2.9%)	0.69	0.10–5.03
*** Burns***	5 (5.1%)	0 (0%)	^*^	^*^
*** Drug use***	32 (32.3%)	20 (29%)	1.17	0.60–2.29

*MSSArs* Medically serious suicide attempters, *ICU* Intensive Care Unit

^*^Statistical comparison tests were not performed due to low n in cross table cells

After multivariate analysis, MSSA needing intensive and specialized care less frequently used self-poisoning and cuts but were more likely to have a life history of mental disorders (see Table 4).

**Table 4:** Multivariate analysis: logistic regression

Variables	*Exp(B)*	Wald	Sig	*Exp(B)* (IC 95%)
Poisoning	8.403	6.965	0.008	(1.73–40.83)
Cuts	5.893	6.555	0.010	(1.52–22.91)
Previous psychiatric disorder	0.274	4.097	0.043	(0.08–0.96)
Constant	1.665	0.671	0.413	

Hosmer and Lemeshow test *X*^*2*^ = 0.55; *p* = 0.46

## Discussion

This is the first study conducted in Spain on MSSA using a highly restrictive definition of a nearly lethal suicide attempt. Notably, the definition of MSSA in our study extended from 24 to 48 h the minimum a patient had to remain hospitalized after the suicide attempt [4, 6–11], so they could be more accurately conceptualized as nearly lethal behaviours (closer to deaths by suicide). In fact, in our study mean hospital stay was 23.68 days thus confirming the severe medical consequences of the attempt. Patients in our sample spent more time hospitalized, more frequently reported recent stressful life events and had higher rates of previous suicide attempts and more frequently reported non-planned suicide attempts. Discordances with other studies could be related to our more restrictive definition of the MSSA. Given the high rate of previous suicide attempts, our sample could be expected to have a high risk of dying by suicide, because as Probert-Lindström's study shows, having previously attempted suicide is a risk factor for death by suicide even decades later [39].

Despite the fact that most MSSA studies assume they are a homogenous group, some authors have found distinctive patterns based on clinical [26] or suicidological features [22]. Although to date few studies have specifically analysed MSSA admitted to an ICU [27, 40], we decided to divide the MSSA sample into two groups as those in intensive care treatment could be categorized as aborted suicides. In fact, MSSArs admitted to ICU are more critically ill as they need to recover from life-threatening conditions.

Hospitalization in an Intensive Care Unit was considered a strong enough criterion to classify MSSA according to their severity as patients admitted to this type of unit are in a more serious and life-threatening condition. A sensibility analysis determining (other) different scenarios for medically serious suicide attempters, using other criteria to assess the seriousness of the attempt such as the score in the Lethality Rating Scale [41], could be considered in future studies.

MSSArs who needed intensive care spent more time in hospital (27.57 days), compared to those admitted to other medical units (18.10 days). Although this difference did not reach statistical significance, which may be due to the small sample size, ICU-admitted MSSA lead to higher costs both from a human and financial point of view.

### Comparison of our sample with other studies on MSSA

No great differences were found in comparison with previous studies on MSSA concerning prevalence of mental disorders (especially affective disorders) and main suicide method (medication overdose). When comparing the profiles of MSSA, women (52.4%) were slightly less represented than Beautrais’ research studies [4, 11], but higher than in others [42]. Deaths by suicide are said to be clearly more frequent among men while non-severe suicide attempts are more frequent among women [43]. MSSA would be in the medium of a continuum of gender-related suicidal behaviours as they are almost equally frequent in both sexes.

Almost all patients met criteria for mental disorders (98.8%), findings that are similar to those reported in other studies on MSSA [4, 6–9, 11, 44]. Affective disorders were also the most frequent diagnosis in our sample [11, 20, 22, 45, 46]

A large number of MSSA in our sample (66.7%) had at least one previous suicide attempt. Our figures were higher compared to other studies [27]. Rapeli and colleagues, when focusing on “aborted suicides”, reported that 59% did not have prior suicide attempts [26].

The most frequent suicide method was medication overdose (67.3%), followed by jumping from heights (23.2%). The results for self-poisoning were similar to other studies but prevalence of jumping was higher than that reported by other studies: 18.6% [18], 14% [19] and 16.7% [21]. Self-immolations amounted to 3% (*n* = 5). Prevalence of this rare condition was higher than in other studies conducted in Western societies [21, 47], but lower than others [17]. Studies conducted in India have registered the highest prevalence of self-immolation 10% (*n* = 10), especially among women, a fact that can be related to certain specific sociocultural factors [23, 24].

Very few studies on MSSA specifically inquire into the combination of different suicide methods. Only one study conducted in a 13–34 years-old sample found that 5% of MSSA were the result of combining several lethal means (i.e.: ingestion, cutting and hanging) [48]. One third of our sample chose more than one method. The most frequent combination was medication overdose and drug use, with alcohol being the most frequent drug of choice. Alcohol would help individuals feel more disinhibited to attempt suicide [48, 49].

In our sample, the overall BIS average score was 26.52, much lower than that identified previously by Giner et al., where the average scores were higher (between 62.5 and 63.3), but similar for the three comparison groups: low lethality suicide attempts, serious suicide attempts and violent suicide attempts [22]. They did not find differences in the Hopelessness Scale between the low lethality SA, serious SA and violent SA subgroups [22], although the average score was similar to our study.

### Comparing MSSA admitted to ICU with other MSSA not admitted to ICU

Subjects with higher educational level were more likely to need admission to ICU after their MSSA. Although there are few studies focusing on MSSA needing admission to ICU, Beautrais, in a 5-year follow-up study, found that subsequent suicide attempts were more frequent among patients with a higher educational level [2]. This finding suggests that individuals with a higher educational level may have more skills to prepare and carry out a more severe lethal attempt. However, other researchers had previously found an association between lower educational levels and MSSA [7, 18, 21, 46] and others failed to find any relationship [6, 19]. Therefore, more insight is needed to ascertain the relationship between socio-educational level and serious suicidal behaviour.

Current suicide attempt medical severity in our study was not related to the presence of a prior physical illness. In fact, no differences in this regard were found between MSSA admitted to ICU and MSSA not admitted to ICU. History of previous suicide attempts were more frequent among MSSArs admitted to ICU compared to those admitted to other medical and surgical units. Although the differences did not remain statistically significant after Bonferroni correction, it is known to be a risk factor that remains even decades after the index attempt [39].

Both patients admitted to ICU and those admitted to other medical and surgical units presented similar SIS scores. Conversely, Rapeli’s study found higher scores on the suicide intentionality subscale amongst the attempted suicides of higher lethality [26], though it cannot be assumed that the higher lethality group were of sufficient medical severity to require ICU admission. This being the case, the differences observed could have resulted from the complex relationship between lethality and intentionality, because the medical severity of an attempt (“objective” lethality) can be independent of the patient’s intention to die by suicide (“subjective” lethality). In this regard, some studies have found a relation between them [24, 50], while others have not been able to confirm it [51].

Other authors refer to some variables modulating the relationship between intentionality and lethality, as is the case of the subject’s prior knowledge about the lethal potential of the method employed. While the expectations of death have been associated with a higher risk of suicide, some authors note their low predictive value [52].

Patients needing ICU admission were less likely to have chosen poisoning and cutting as their suicide method and were more likely to have had prior mental health problems, compared to those subjects admitted to other medical units. Attempters admitted to ICU more frequently reported a history of prior mental disorders but only Beautrais [6–8] reported that patients with MSSA were more likely to have a prior history of affective disorders compared to controls and to those who had died by suicide.

## Limitations

The main limitations of this study are: a) its cross-sectional design that limits the knowledge of risk and protective factors related to MSSA; b) the sample size, especially in the subsample analysis; c) the fact that diagnoses were not obtained after a structured interview; and, d) the lack of more qualitative and phenomenological information with respect to MSSA.

The lack of a structured diagnostic interview might have influenced the results as there may be a moderate correlation between diagnoses conducted with and without those types of instruments [53, 54]. However, individuals requiring hospitalization after an MSSA usually have serious injuries related to the suicide attempt limiting the feasibility to carry out a long semi-structured diagnostic interview psychometric assessment.

## Conclusions

Despite its limitations, data on highly severe MSSA needing intensive and specialized care treatment (admission in ICU) reported in this study may help improve strategies aimed at preventing future suicides. In situ evaluation of patients after an MSSA may provide a valuable insight into the factors and reasons behind their behaviour. New ways of exploring the mental states leading to severe attempts should be incorporated in future research.

Preventing MSSA should be considered a public health priority in order to reduce the negative impact on the patient’s wellbeing and also to lessen the healthcare system’s expenditure on this type of suicidal behaviours. Findings from longitudinal studies on nearly lethal suicide attempts may enrich the conclusions from this study. Qualitative studies may also contribute to a wider comprehension of the phenomenon.

## Data Availability

The data of the current study are not publicly available due to their containing information that could compromise the privacy of the participants. The anonymized data and the research protocol that support the findings are available on request from the corresponding author, MQ-F.

## Abbreviations

MSSA: Medically serious suicide attempts
MSSArs: Medically serious suicide attempters
ICU: Intensive Care Units
SA: Suicide attempt
Index: Suicide Attempt (ISA)
